# Supplementary material for: Reconstructing the regulatory programs underlying the phenotypic plasticity of neural cancers
Source: Nat Commun. 2024 Nov 9;15:9699. doi: 10.1038/s41467-024-53954-3 (PMC11549355; doi:10.1038/s41467-024-53954-3)
Supplement: Supplementary file 1 — Supplementary Information [file 41467_2024_53954_MOESM1_ESM.pdf]

Supplementary Material: Reconstructing the regulatory programs underlying the phenotypic plasticity of neural cancers

Larsson, Held et al.

Supplementary Note 1: Generation of data-driven simulated data

We performed data-driven simulation to investigate our algorithm and to demonstrate various decision tools introduced in the main text. The PBMC 3k dataset used in the manuscript was first preprocessed using *sctransform* version 2 [1]. Let x_{jl} be the observed count of gene j in cell l. During variance stabilisation, *sctransform* computes total UMI counts $n_l = \sum_j x_{jl}$ and performs general linear regression with negative binomial error model, using the linear predictor $\beta_j + \log(n_l)$ and dispersion parameter θ_j. Stabilised estimates of intercept β_j and dispersion parameter θ_j can then be extracted from the *sctransform* fit. In addition, we use the Pearson residuals z_{jl} of the 2000 most variable genes obtained from *sctransform*. Among these genes 150 are transcription factors (TF).

We construct a ground-truth containing 4 clusters with 47, 59, 132, and 32 genes each, in addition, 1578 are marked as noise. These clusters are associated with 7 to 10 regulators each and coefficients $\mathbf{B}_i$ for cluster i are set to values comparable with results obtained from applying *scregclust* on the data. This ensures that effect sizes are comparable to real data runs. New count data is then simulated using the negative binomial distribution with gene- and cell-dependent mean value and dispersion. New gene counts in the rag bag are simulated as

$$y_{jl} \sim \text{Neg-Binom}(n_l \exp(\beta_j), \theta_j)$$

and gene counts in clusters are simulated as

$$y_{jl} \sim \text{Neg-Binom}(n_l \exp\left(\beta_j + \mathbf{Z}^{(l,\mathrm{R}_i)}\mathbf{B}_i^{(:,j)}\right), \theta_j).$$

Supplementary Note 2: Choosing hyperparameters

The *scregclust* algorithm involves several hyperparameters, as listed on `https://scmethods.github.io/scregclust/reference/scregclust.html`. For the majority of these, default values are provided and the typical user will not need to make any changes to these. For a more thorough mathematical background to these hyperparameters, we refer to the section "Clustering algorithm" in Methods.

For some of the hyperparameters, a user-defined input is however required (`penalization`, `n_modules`) or strongly recommended (`min_module_size`). To facilitate for the user in running the algorithm, we have compiled a comprehensive guide on how to choose values for these key hyperparameters. Based on the analyses made on real data in this paper, we also provide a recommended range for these parameters, as a starting point to choosing these for the user's specific case.

- `penalization`: a sparsity penalty controlling the amount of regulators used for each cluster. The recommended strategy to choose the penalty parameter has been thoroughly described and exemplified in the section "Regulatory-driven clustering: performance and robustness" and Fig. 2, both in the main manuscript. The strategy involves testing a range of penalty parameters and finding the most appropriate one using the metrics "predictive R^2 per module" and "regulator importance". In the vignette accompanying the algorithm, an example on real data (the PBMC dataset) on how to choose this parameter is included. Recommended range: At least ten different penalty parameters between 0.01 and 0.05.

- `n_modules`: requested number of target modules. Similar to clustering algorithms like kmeans, this hyperparameter controls the number of target gene modules, with the important difference that *scregclust* can produce empty clusters if the value for this parameter is set too high. To choose an appropriate value, we have introduced silhouette scores, as described in the section "Regulatory-driven clustering: performance and robustness" in the main manuscript, as well as in Supplementary Fig. 1. Recommended range: 10-20.

- `min_module_size`: minimum required number of target genes in a module. The default value for this parameter is 0, leading to it having no effect. However, a gene module consisting of few genes might not be meaningful as a signature of a cell type or biological process, and we therefore recommend a value of 20 for this parameter.

In addition to above hyperparameters, the user could consider changing the value for `noise_threshold`. This parameter controls the threshold for the best R^2 of a target gene before it gets identified as noise, and placed in the rag bag cluster. Currently, the default value is 0.025, which is quite stringent with the consequence being a substantial amount of genes in the rag bag cluster. Increasing or decreasing this parameter will affect the size of the rag bag cluster.

Supplementary Note 3: Comparison with other methods

To the best of the authors' knowledge, no method directly comparable to *scregclust* exists. In addition to comparing to SCENIC+ [2] in the main manuscript, we therefore compared different aspects of *scregclust* with a selection of other methods, where comparable. All evaluations were performed on real data, specifically on the glioblastoma scRNA-seq dataset by Wang *et al.* [3] and the PBMC scRNA-seq dataset used in the main manuscript for comparison with SCENIC+.

The following three aspects were evaluated:

1. **Cluster stability (Supplementary Fig. 1)**: We compared the gene clustering from *scregclust* to gene clusterings obtained directly on the target genes using three gene clustering methods, two traditional methods (k-means and hierarchical clustering with cosine angle as a distance measure and complete linkage) and one method specifically developed for scRNA-seq data (Celda [4]). For all clustering methods, a range of clusters from 2 to 20 was requested. Clustering stability was measured by Adjusted Rand Index; genes were randomly broken into three groups. Each algorithm was run on Part 1 + 3 and then Part 2 + 3. Stability of clustering was evaluated by comparing the clustering of Part 3 between the two runs repeated 50 times. In this comparison, hierarchical clustering consistently performed the worst while kmeans, Celda and *scregclust* showed comparable performance. For small cluster numbers, kmeans displayed slightly better cluster stability but this dropped and got more variable with increasing number

of clusters, sometimes performing substantially worse than *scregclust*. Celda demonstrated a variable performance across all cluster numbers, while *scregclust* had a stable behavior throughout runs and cluster number.

2. **Regulator stability (Supplementary Figs. 2–6)**: *scregclust*'s ability to detect regulators consistently across repeated runs as well as its ability to find a relevant amount of regulators was evaluated against two established network methods (PPCOR [5] and WGCNA [6]) combined with community detection, a variant of WGCNA adapted to single-cell data, hdWGCNA [7], as well as against GRNboost2 [8] coupled with kmeans clustering or Celda and finally PIDC [9] coupled with Celda, the last combination being an example of two state-of-the-art methods for scRNA-seq data. hdWGCNA, Celda, GRNboost2 (when combined with Celda), and PIDC are run on counts. The rationale for combining gene regulatory network methods with gene clustering methods is that *scregclust* is designed to avoid creating a complete network with an unfeasibly high number of parameters, focusing instead on how key regulators affect major cellular processes. Therefore, direct comparison with gene regulatory network methods would have been out of scope.

   The stability metric was computed by finding all edges between regulators and genes in Part 3 above predicted by a method, each method for a range of parameters, and computing the proportion of times across 50 runs a regulator is associated with at least one of the modules. This was then plotted against the tuning parameters (*scregclust* penalty, WGCNA/hdWGCNA power and PPCOR threshold) (Supplementary Figs. 2 and 3). Briefly, *scregclust* (a) is more stable than WGCNA/hdWGCNA (c,e) as indicated by the number of regulators that drop off as we change the penalty (truncated lines). It also detects around twice as many regulators (b,d,f). PPCOR appear sensitive to the choice of threshold and rapidly goes from selecting most regulators to almost none (g,h). Applied to the Wang et al data, WGCNA showed inconsistent variable selection for different parameters (Supplementary Fig. 2). By construction, regulator identification with GRNboost2 and PIDC is conducted separately for each target gene. Thus, when we cluster target genes there is no guarantee that the same regulators are selected for all targets in the same cluster. Here, we compute a stability metric from the proportion of target genes in a cluster that share a selected regulator (Supplementary Figs. 4–6). For perfect stability, this proportion should be 1, meaning all target genes share the same regulator. Crucially, by construction, *scregclust* achieves a per-cluster regulator stability of 1.

3. **Regulator sign-consistency (Supplementary Figs. 7–8)**: One of *scregclust*'s major strengths is the estimation of sign-consistent regulatory effect within modules. To assess how other methods perform, we calculated the signs of the regulatory effects on genes in Part 3. Because one regulator can affect multiple genes in a cluster, we examined the distribution of signs for that regulator within the cluster. We then compute the proportion of the most common sign (+1 or -1). This is then compared against the number of connections to that regulator in the cluster. In *scregclust*, this process is integrated into the method, ensuring that sign consistency within clusters is always achieved with a majority sign proportion of 1. Other methods (PPCOR, WGCNA, hdWGCNA, kmeans + GRNBoost2, Celda + GRNBoost2, Celda + PIDC) do not have a straightforward way of doing this. One approach used in other methods, such as SCENIC+, is to consider the sign of the marginal Spearman correlation between a regulator and a gene. This was the method used for the approaches we compared (Supplementary Figs. 7 and 8). However, it is clear that these methods do not guarantee sign consistency within clusters, meaning target clusters contain genes that are not regulated by the identified regulators in the same way.

Supplementary figures

(a) PBMC (b) Wang et al. (2019) [3]

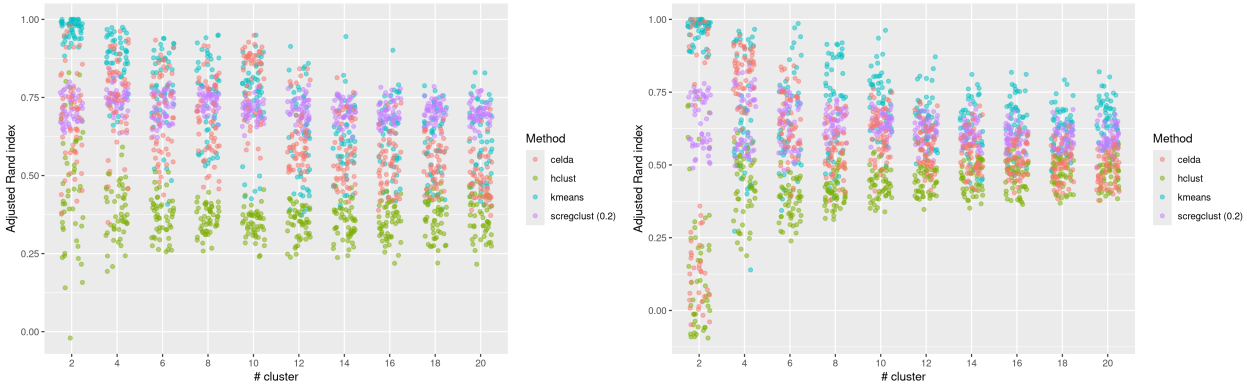

Supplementary Figure 1 Cluster stability. *scregclust* is stable over multiple runs and for a wide range of number of clusters. k-means is stable for a smaller number of clusters but exhibits a large variation across multiple runs, sometimes performing substantially worse than *scregclust*, a behavior also seen with Celda. Hierarchical clustering performs worse overall. (a) Results for PBMC data. (b) Results for Wang et al. (2019) data

(a) scregclust, stability

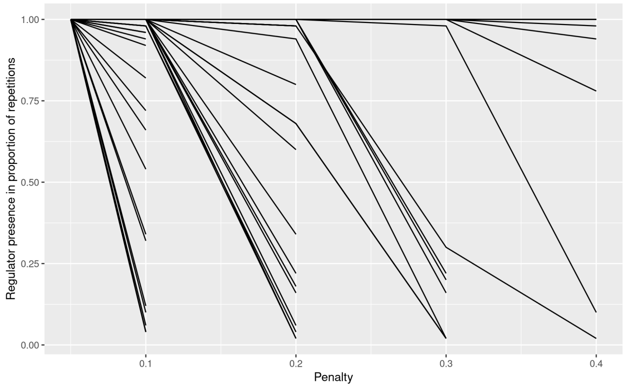

(b) scregclust, # regulators

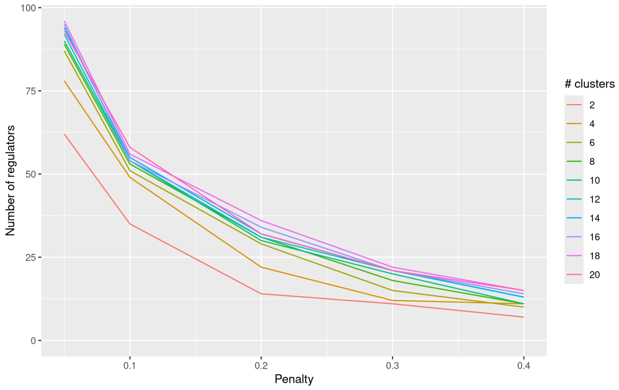

(c) WGCNA, stability

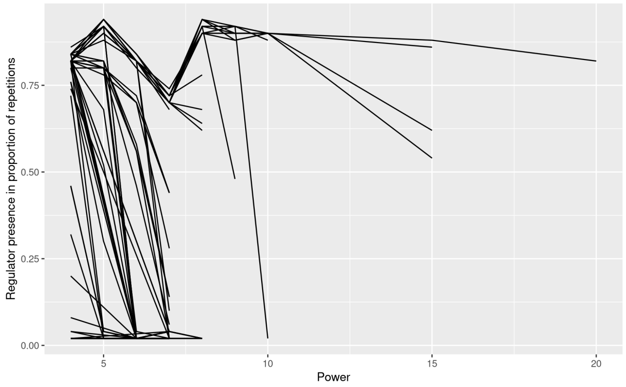

(d) WGCNA, # regulators

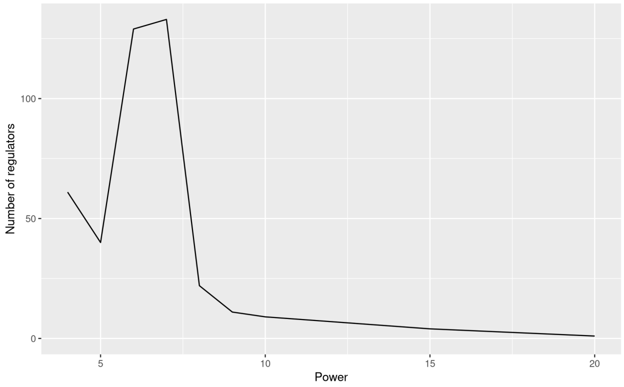

(e) hdWGCNA, stability

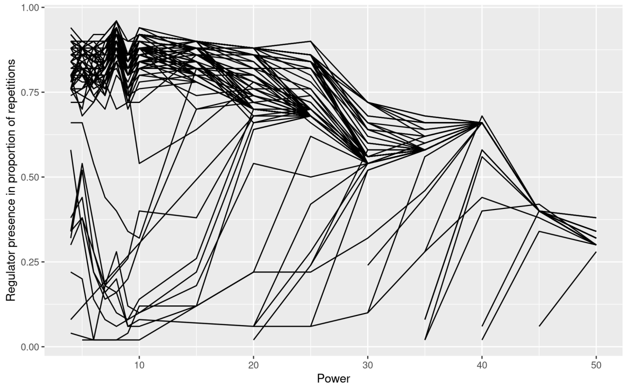

(f) hdWGCNA, # regulators

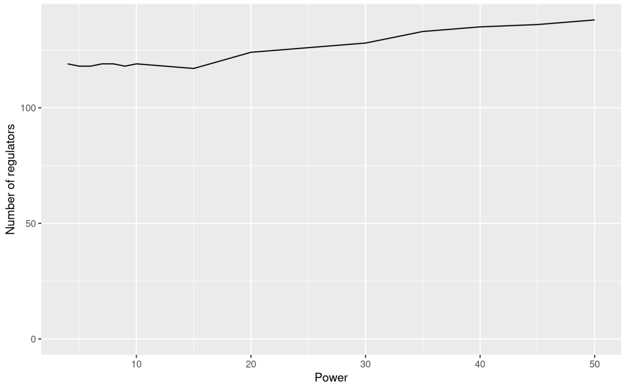

(g) PPCOR, stability

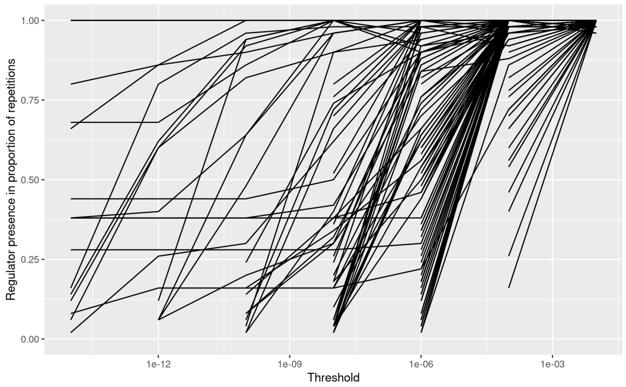

(h) PPCOR, # regulators

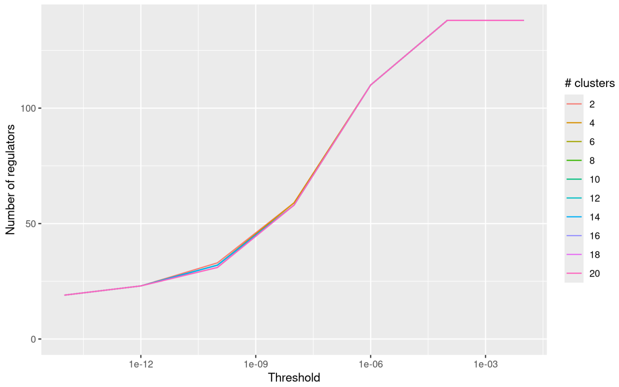

Supplementary Figure 2 Regulator stability assessed on the Wang et al. (2019) data set. We plot regulator stability across 50 repetitions as a function of the method specific tuning parameter (penalty, power, threshold) for (a) scregclust, (c) WGCNA, (e) hdWGCNA, and (g) PPCOR and the total number of regulators selected in each setting (in (b), (d), (f), and (h) corresponding to the same methods in the left column). The selection profiles, one black curve for each regulator (in figures (a), (c), (e), and (g))), should ideally exhibit a high presence for a range of penalties. For this data set, scregclust (a, shown for # clusters = 8) is clearly more stable than WGCNA (c) and detects around twice as many regulators. hdWGCNA (e) exhibits poor performance. PPCOR (g, shown for # clusters 8) performs better on this data set but is still sensitive to the choice of threshold.

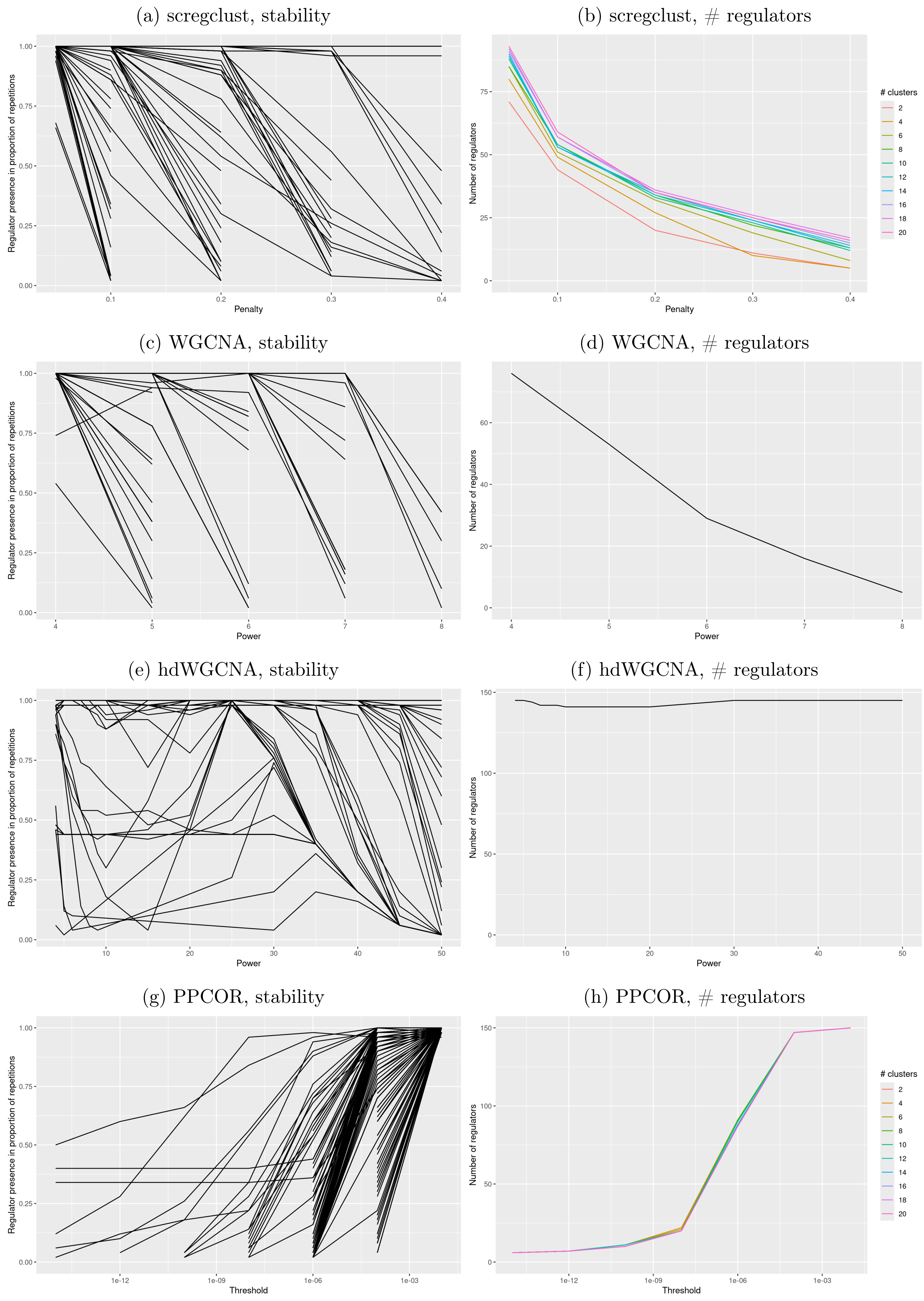
(a) scregclust, stability
(b) scregclust, # regulators
(c) WGCNA, stability
(d) WGCNA, # regulators
(e) hdWGCNA, stability
(f) hdWGCNA, # regulators
(g) PPCOR, stability
(h) PPCOR, # regulators
Regulator presence in proportion of repetitions
Number of regulators
Penalty
Power
Threshold
clusters
2
4
6
8
10
12
14
16
18
20

Supplementary Figure 3 Regulator stability, assessed on the PBMC data set. We plot regulator stability as a function of the method specific tuning parameter (penalty, power, threshold) for (a) scregclust, (c) WGCNA, (e) hdWGCNA, and (g) PPCOR across 50 repetitions, and the total number of regulators selected in each setting (in (b), (d), (f), and (h) corresponding to the same methods in the left column). The selection profiles, one black curve for each regulator (in figures (a), (c), (e), and (g)), should ideally exhibit a high presence for a range of penalties. scregclust (a, results shown for # clusters = 8) is more stable than WGCNA (c) as indicated by the number of regulators that drop off as we change the penalty (truncated lines) and detects around twice as many regulators. hdWGCNA (e) is not stable. PPCOR (g, shown for # clusters = 8f) is very sensitive to the choice of threshold and rapidly goes from selecting most regulators to almost none.

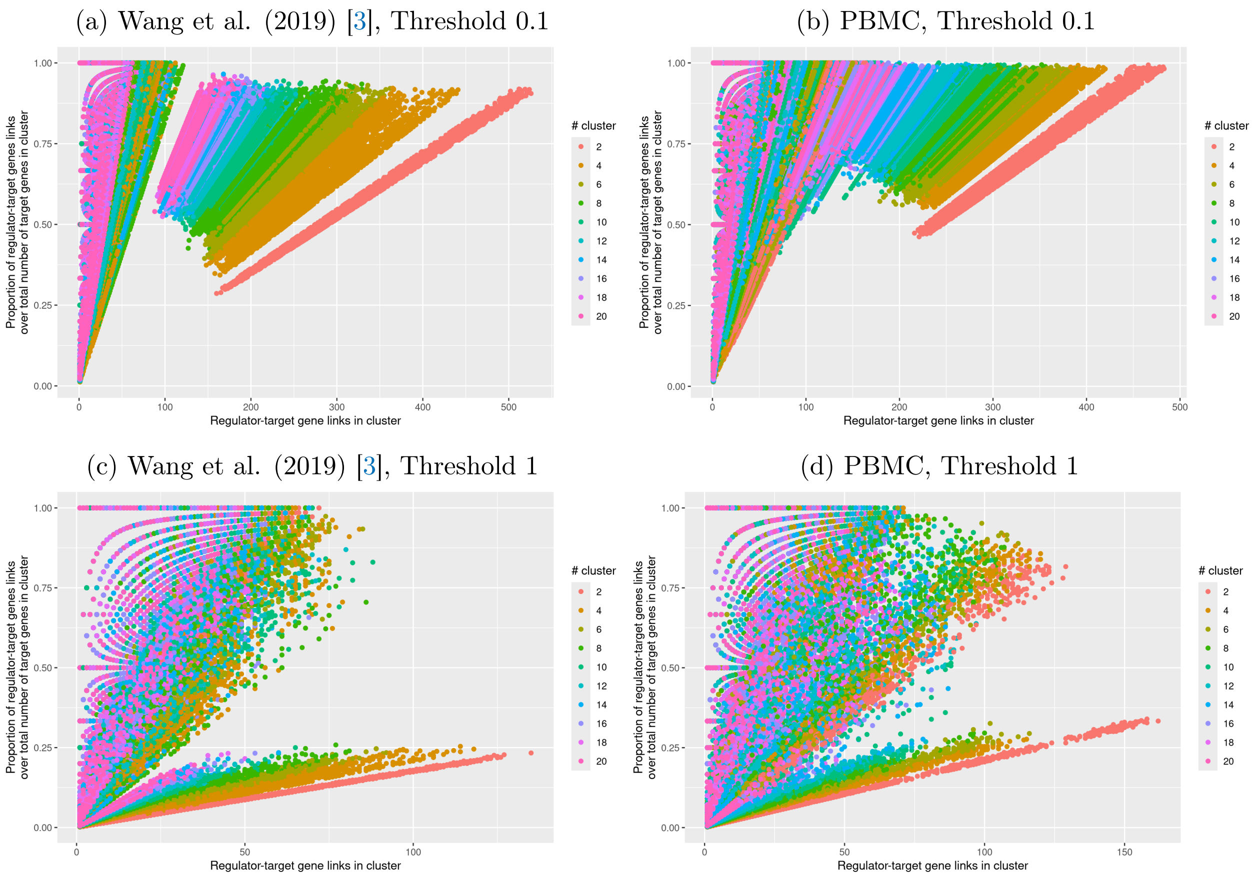

Supplementary Figure 4 Regulator stability for kmeans + GRNboost2 Each dot represents a regulator. On the x-axis, the number of target genes associated with this regulator is shown. The y-axis indicates the proportion of target genes in a cluster that also is associated with this regulator, colored by the number of clusters. Since GRNboost2 selects regulators on a gene-by-gene basis, selection consistency across genes in a cluster is not guaranteed. scregclust results in perfectly consistent (proportion 1) results by construction. kmeans+GRNboost2 exhibits a wide range of identified regulators for every target gene in a cluster for both data sets and for different tuning parameter settings (threshold). These results support the need for a joint estimation procedure. (a) and (c) show results for the Wang et al. (2019) data. (b) and (d) show results for the PBMC data. In (a) and (b) results for threshold 0.1 are shown. In (c) and (d) results for threshold 1 are shown.

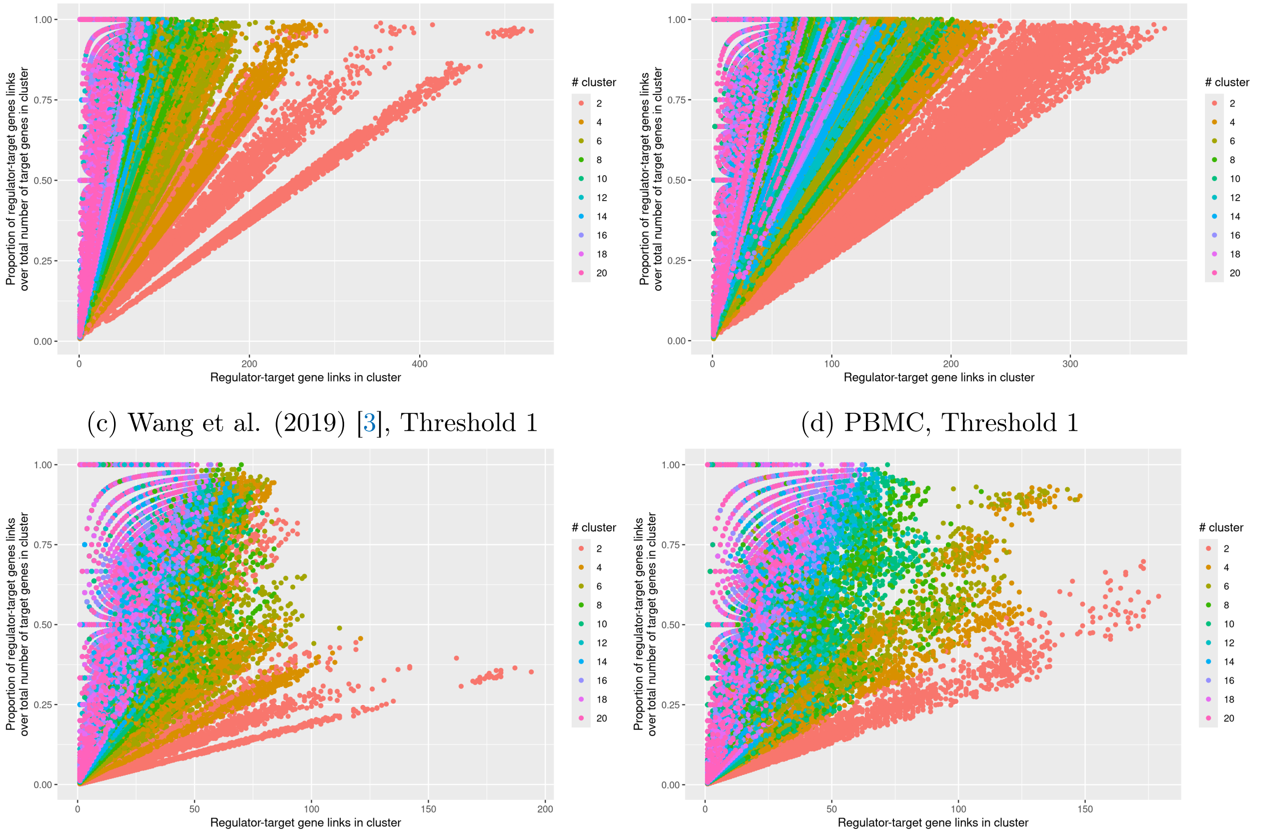

(a) Wang et al. (2019) [3], Threshold 0.1

(b) PBMC, Threshold 0.1

(c) Wang et al. (2019) [3], Threshold 1

(d) PBMC, Threshold 1

Supplementary Figure 5 Regulator stability for Celda + GRNboost2 Each dot represents a regulator. On the x-axis, the number of target genes associated with this regulator is shown. The y-axis indicates the proportion of target genes in a cluster that also is associated with this regulator, colored by the number of clusters. Since GRNboost2 selects regulators on a gene-by-gene basis, selection consistency across genes in a cluster is not guaranteed. scregclust results in perfectly consistent (proportion 1) results by construction. Celda+GRNboost2 exhibits a wide range of identified regulators for every target gene in a cluster for both data sets and for different tuning parameter settings (threshold). These results support the need for a joint estimation procedure. (a) and (c) show results for the Wang et al. (2019) data. (b) and (d) show results for the PBMC data. In (a) and (b) results for threshold 0.1 are shown. In (c) and (d) results for threshold 1 are shown.

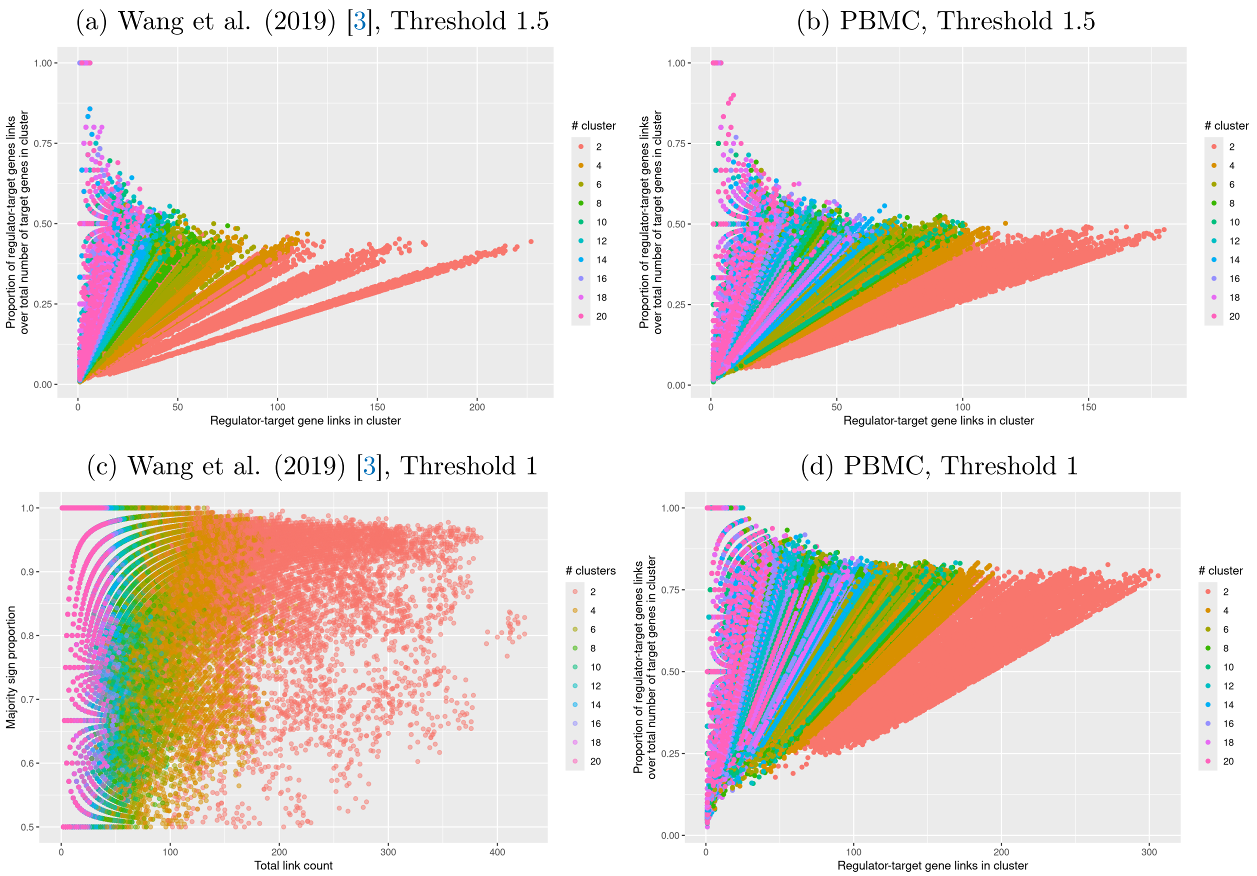

Supplementary Figure 6 Regulator stability for Celda + PIDC Each dot represents a regulator. On the x-axis, the number of target genes associated with this regulator is shown. The y-axis indicates the proportion of target genes in a cluster that also is associated with this regulator, colored by the number of clusters. Since PIDC selects regulators on a gene-by-gene basis, selection consistency across genes in a cluster is not guaranteed. *scregclust* results in perfectly consistent (proportion 1) results by construction. Celda+PIDC exhibits a wide range of identified regulators for every target gene in a cluster for both data sets and for different tuning parameter settings (threshold). These results support the need for a joint estimation procedure. (a) and (c) show results for the Wang et al. (2019) data. (b) and (d) show results for the PBMC data. In (a) and (b) results for threshold 1.5 are shown. In (c) and (d) results for threshold 1 are shown.

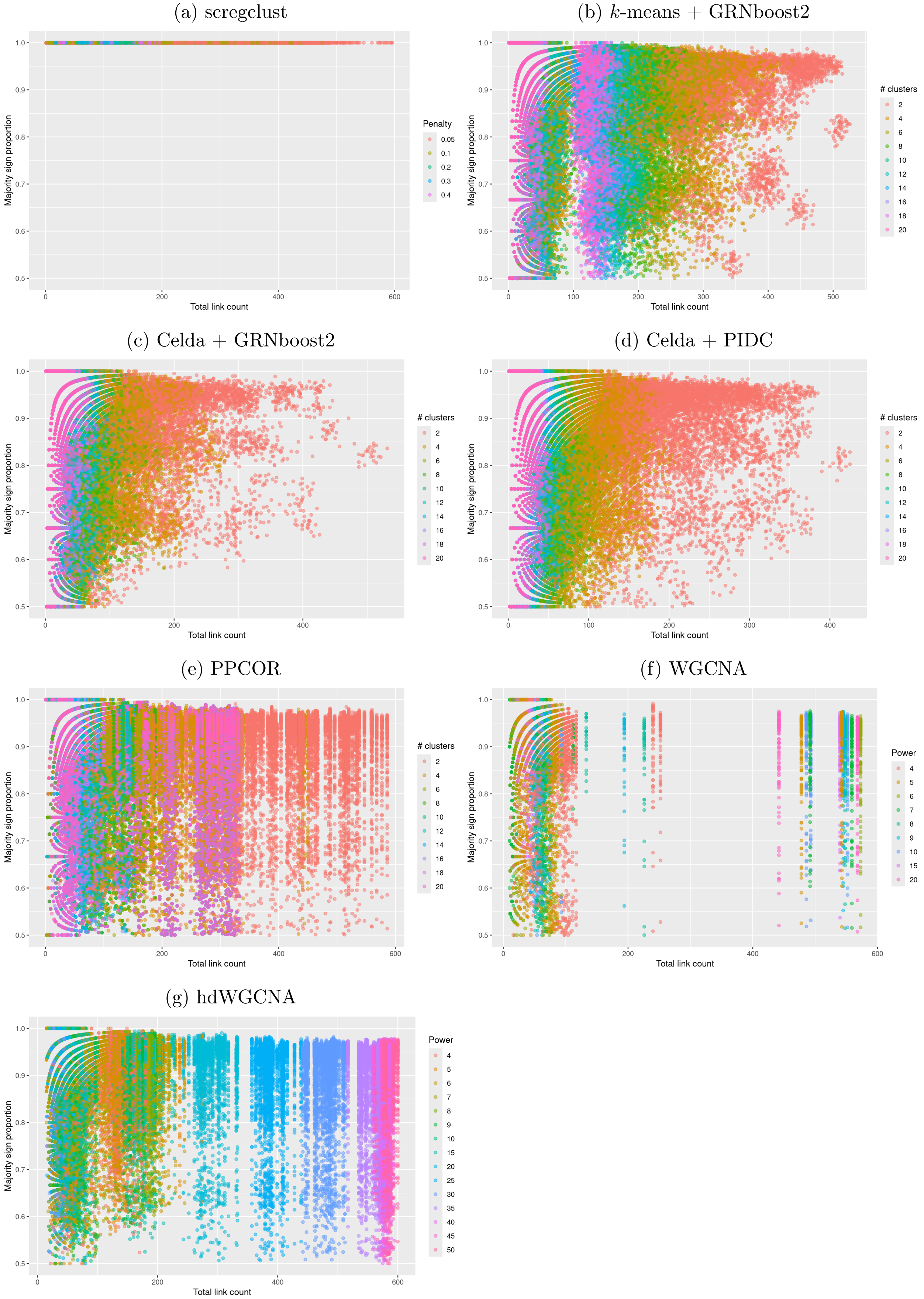
(a) scregclust
Majority sign proportion
Total link count
Penalty
0.05
0.1
0.2
0.3
0.4
(b) k-means + GRNboost2
clusters
2
4
6
8
10
12
14
16
18
20
(c) Celda + GRNboost2
(d) Celda + PIDC
(e) PPCOR
(f) WGCNA
Power
4
5
6
7
8
9
10
15
20
(g) hdWGCNA
25
30
35
40
45
50

Supplementary Figure 7 Sign consistency assessed on the Wang et al. (2019) data set We plot the proportion of the most common sign (+1 or -1) for each regulator per cluster for (a) *scregclust*, (b) k-means + GRNboost2, (c) Celda + GRNboost2, (d) Celda + PIDC, (e) PPCOR, (f) WGCNA, and (g) hdWGCNA against the number of target genes in the cluster that the regulator is linked to. By construction, scregclust is sign-consistent. The majority of methods/method combinations exhibit poor performance. WGCNA is somewhat better but still results in a wide range of results across regulators, ranging from random sign (0.5) to consistent (1).

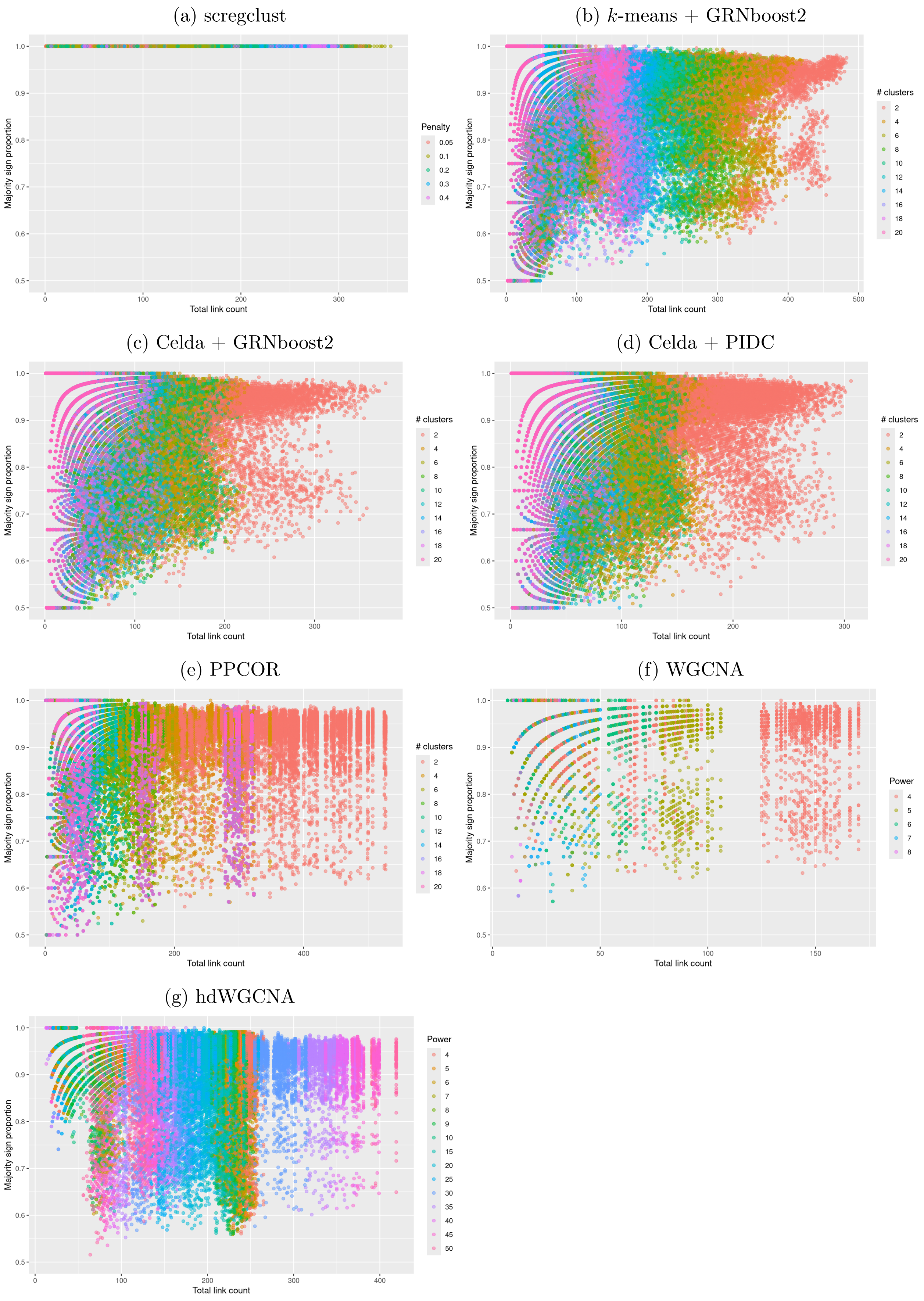
(a) scregclust
(b) k-means + GRNboost2
(c) Celda + GRNboost2
(d) Celda + PIDC
(e) PPCOR
(f) WGCNA
(g) hdWGCNA
Majority sign proportion
Total link count
Penalty
clusters
Power

Supplementary Figure 8 Sign consistency assessed on the PBMC data set. We plot the proportion of the most common sign (+1 or -1) for each regulator per cluster for (a) *scregclust*, (b) k-means + GRNboost2, (c) Celda + GRNboost2, (d) Celda + PIDC, (e) PPCOR, (f) WGCNA, and (g) hdWGCNA against the number of target genes in the cluster that the regulator is linked to. By construction, scregclust is sign-consistent. The majority of methods/method combinations exhibit poor performance. WGCNA is somewhat better but still results in a wide range of results across regulators, ranging from random sign (0.5) to consistent (1).

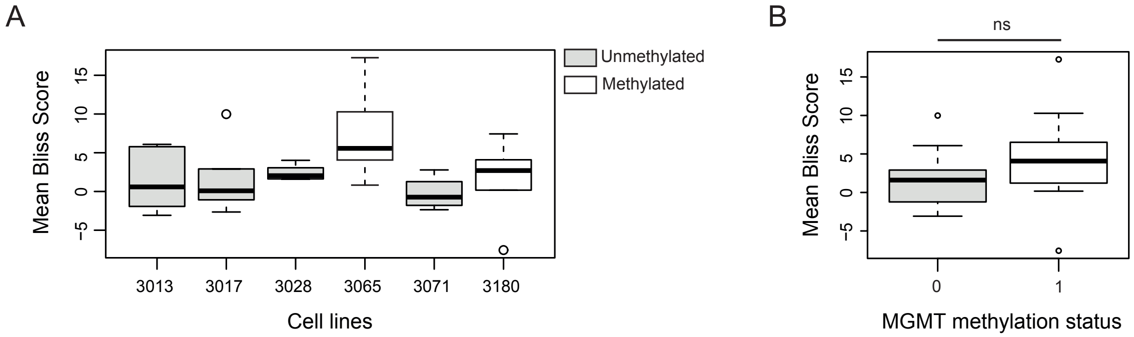

Supplementary Figure 9 (A) Mean bliss score per cell line, MGMT methylation status indicated by grey (unmethylated) or white (methylated). For each condition (cell line) three biological replicates were performed (n=3). (B) Mean bliss score, separated by cell line methylation status, significance testing using Student's t-Test, p-value = 0.1252. Boxplots consist of center lines (median), box bounds (1st and 3rd quartile), and upper and lower whiskers. Upper whiskers are drawn from the upper box bound to the largest data point but no further than 1.5 times the inter-quartile range (IRQ), analogous for lower whiskers. All data points not covered by box and whiskers are shown as dots.

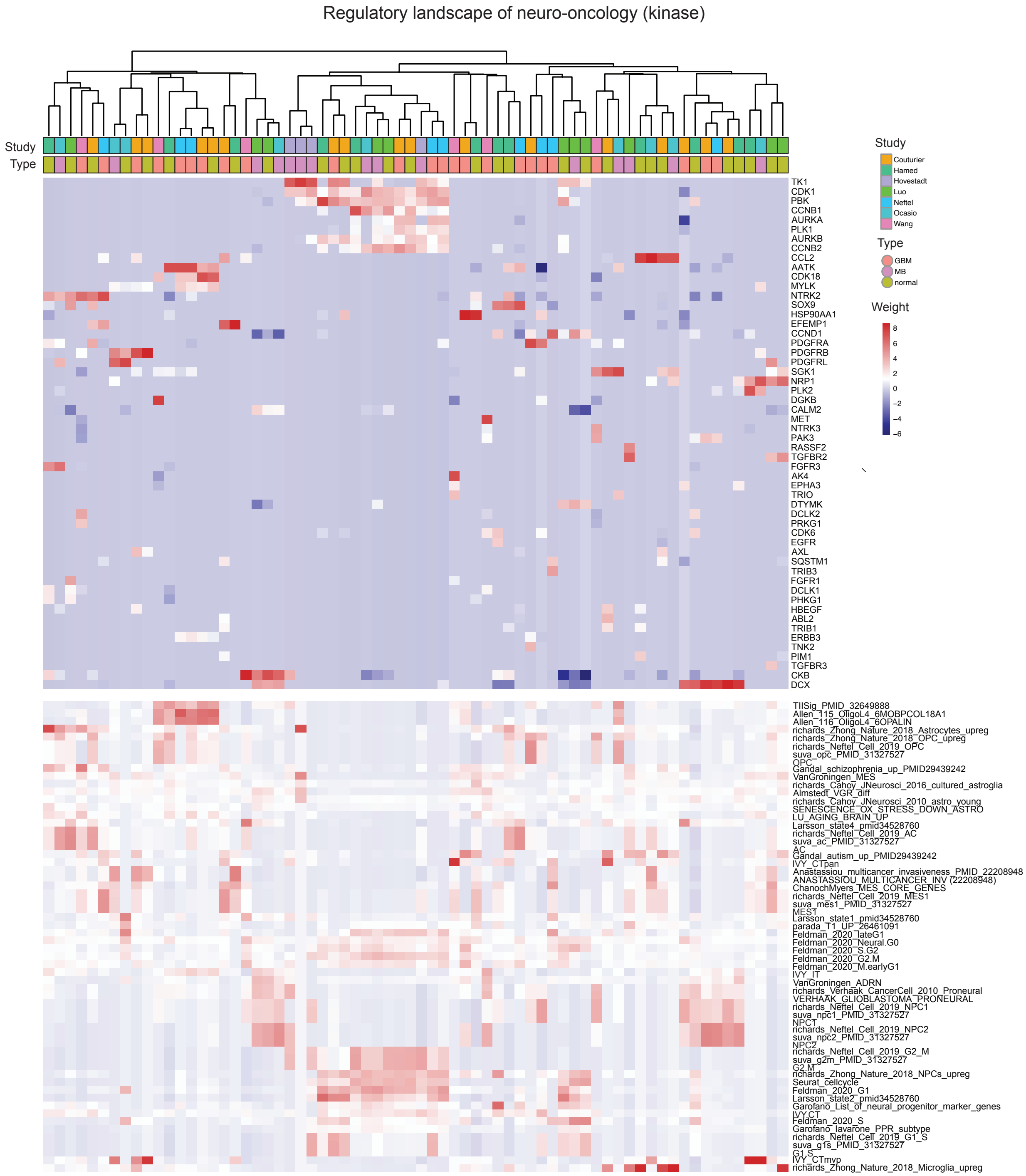

Supplementary Figure 10 The regulatory landscape of neuro-oncology - kinase version. The figure is analogous to Fig. 4A, but here the algorithm has been run in kinase mode and excluding neuroblastoma-samples. Middle panel is the regulatory table from *scregclust*, with modules as columns and regulators (kinases) as rows. Top panel are annotation bars indicating what cancer type and study each module originate from. Bottom panel display enrichments for each module against a database of neuro-oncology related gene sets.

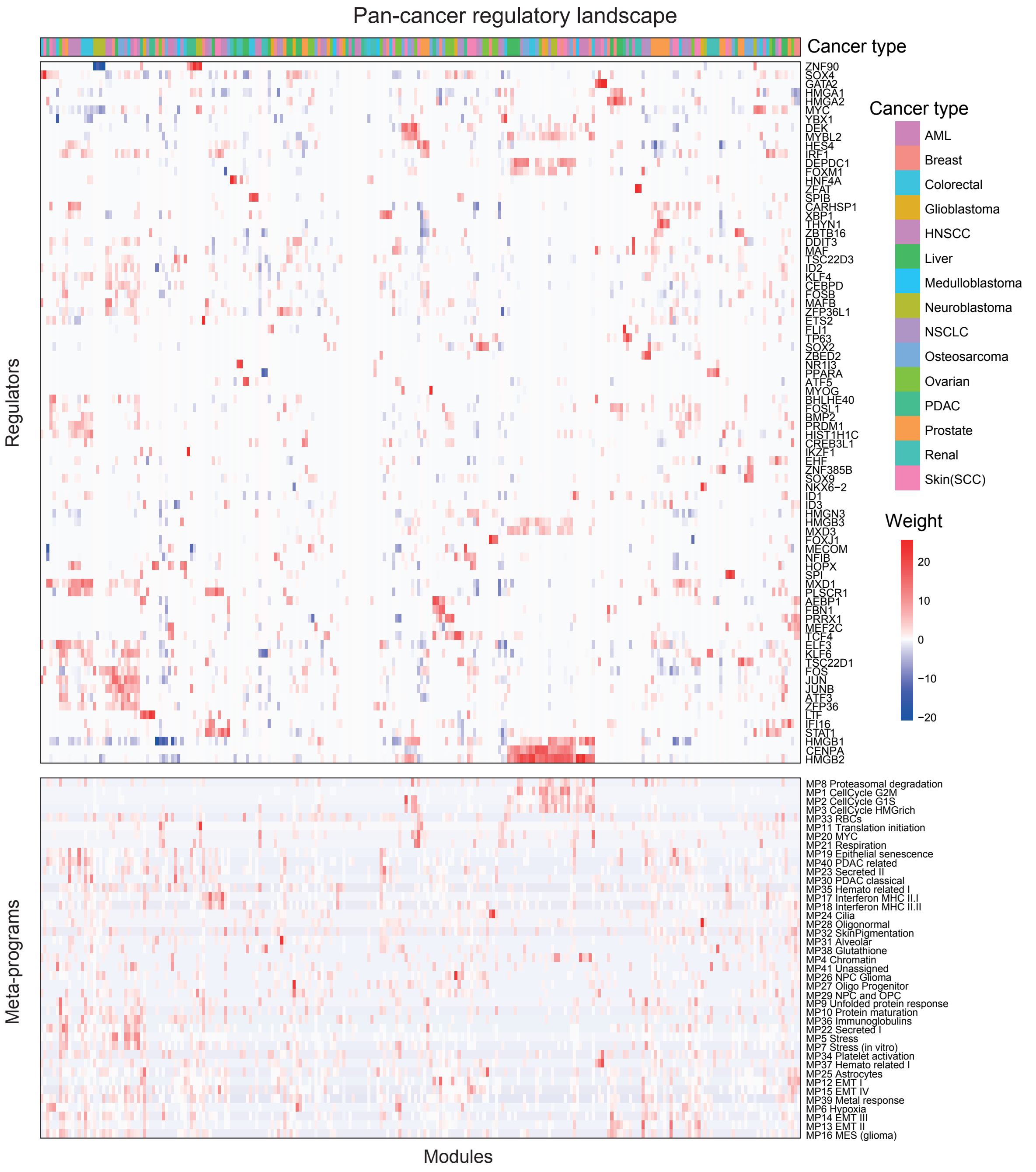

Supplementary Figure 11 Pan-cancer regulatory landscape. This figure is related to Fig. 5. *scregclust* has been run on 15 datasets from 15 different tumor types. Middle panel is the merged regulatory table from *scregclust*, with modules as columns and regulators (transcription factors) as rows. Top panel are annotation bars indicating what cancer type each module originate from. Bottom panel display enrichments for each module against gene signatures of hallmarks of intratumoral heterogeneity in cancer, from [10].

Supplementary References

1. Choudhary, S. & Satija, R. Comparison and evaluation of statistical error models for scRNA-seq. *Genome Biol.* **23** (2022).
2. Bravo González-Blas, C. *et al.* SCENIC+: single-cell multiomic inference of enhancers and gene regulatory networks. *Nat. Methods* **20,** 1355–1367 (2023).
3. Wang, L. *et al.* The phenotypes of proliferating glioblastoma cells reside on a single axis of variation. *Cancer Discov.* **9,** 1708–1719 (2019).
4. Wang, Z. *et al.* Celda: a Bayesian model to perform co-clustering of genes into modules and cells into subpopulations using single-cell RNA-seq data. *NAR Genom. Bioinform.* **4,** lqac066 (2022).
5. Kim, S. ppcor: An R Package for a Fast Calculation to Semi-partial Correlation Coefficients. *Commun. Stat. Appl. Methods* **22,** 665–674 (2015).
6. Langfelder, P. & Horvath, S. WGCNA: an R package for weighted correlation network analysis. *BMC Bioinform.* **9,** 559 (2008).
7. Morabito, S., Reese, F., Rahimzadeh, N., Miyoshi, E. & Swarup, V. hdWGCNA identifies co-expression networks in high-dimensional transcriptomics data. *Cell Rep. Methods* **3,** 100498 (2023).
8. Moerman, T. *et al.* GRNBoost2 and Arboreto: efficient and scalable inference of gene regulatory networks. *Bioinformatics* **35,** 2159–2161 (2019).
9. Chan, T. E., Stumpf, M. P. & Babtie, A. C. Gene Regulatory Network Inference from Single-Cell Data Using Multivariate Information Measures. *Cell Syst.* **5,** 251–267.e3 (2017).
10. Gavish, A. *et al.* Hallmarks of transcriptional intratumour heterogeneity across a thousand tumours. *Nature* **618,** 598–606 (2023).
