# Supplementary material for: Reconstructing the regulatory programs underlying the phenotypic plasticity of neural cancers
Source: Nat Commun. 2024 Nov 9;15:9699. doi: 10.1038/s41467-024-53954-3 (PMC11549355; doi:10.1038/s41467-024-53954-3)
Supplement: Supplementary file 3 — Description of Additional Supplementary Files [file 41467_2024_53954_MOESM3_ESM.pdf]

Description of Additional Supplementary Files

Supplementary Data 1: Human Protein Atlas annotation of non-overlapping regulators between scregclust and SCENIC+, related to Figs. 1D-E.

Supplementary Data 2: Regulator x meta-program analysis.

Supplementary Data 3: Hyperparameters used for all scregclust runs in the main manuscript.
