# Supplementary material for: Reconstructing the regulatory programs underlying the phenotypic plasticity of neural cancers
Source: Nat Commun. 2024 Nov 9;15:9699. doi: 10.1038/s41467-024-53954-3 (PMC11549355; doi:10.1038/s41467-024-53954-3)
Supplement: Supplementary file 7 — Reporting Summary [file 41467_2024_53954_MOESM7_ESM.pdf]

Corresponding author(s): Sven Nelander
Last updated by author(s): Oct 14, 2024

Reporting Summary

Nature Portfolio wishes to improve the reproducibility of the work that we publish. This form provides structure for consistency and transparency in reporting. For further information on Nature Portfolio policies, see our Editorial Policies and the Editorial Policy Checklist.

Statistics

For all statistical analyses, confirm that the following items are present in the figure legend, table legend, main text, or Methods section.

n/a	Confirmed	
☐	☒	The exact sample size (n) for each experimental group/condition, given as a discrete number and unit of measurement
☐	☒	A statement on whether measurements were taken from distinct samples or whether the same sample was measured repeatedly
☐	☒	The statistical test(s) used AND whether they are one- or two-sided<br>*Only common tests should be described solely by name; describe more complex techniques in the Methods section.*
☐	☒	A description of all covariates tested
☐	☒	A description of any assumptions or corrections, such as tests of normality and adjustment for multiple comparisons
☐	☒	A full description of the statistical parameters including central tendency (e.g. means) or other basic estimates (e.g. regression coefficient) AND variation (e.g. standard deviation) or associated estimates of uncertainty (e.g. confidence intervals)
☐	☒	For null hypothesis testing, the test statistic (e.g. F, t, r) with confidence intervals, effect sizes, degrees of freedom and P value noted<br>*Give P values as exact values whenever suitable.*
☒	☐	For Bayesian analysis, information on the choice of priors and Markov chain Monte Carlo settings
☒	☐	For hierarchical and complex designs, identification of the appropriate level for tests and full reporting of outcomes
☐	☒	Estimates of effect sizes (e.g. Cohen's d, Pearson's r), indicating how they were calculated

Our web collection on statistics for biologists contains articles on many of the points above.

Software and code

Policy information about availability of computer code

Data collection	Raw counts were downloaded from the indicated source in Table 1 in Methods.
Data analysis	Data analysis was performed in R (v4.4.1) using the following packages: Seurat (v.5.1.0), sctransform (v.0.4.1), Rcpp (v.1.0.13), RcppEigen (v0.3.4.0.2), igraph (v2.0.3) and ggplot2 (v3.5.1). In addition, data analysis was performed using our custom software, scregclust v0.1.9, available at GitHub (https://github.com/scmethods/scregclust).

For manuscripts utilizing custom algorithms or software that are central to the research but not yet described in published literature, software must be made available to editors and reviewers. We strongly encourage code deposition in a community repository (e.g. GitHub). See the Nature Portfolio guidelines for submitting code & software for further information.

Data

Policy information about availability of data

All manuscripts must include a data availability statement. This statement should provide the following information, where applicable:

- Accession codes, unique identifiers, or web links for publicly available datasets
- A description of any restrictions on data availability
- For clinical datasets or third party data, please ensure that the statement adheres to our policy

No new datasets were generated for this paper. The publicly available data used in this study are available in the following repositories: The Curated Cancer Cell Atlas \url{https://www.weizmann.ac.il/sites/3CA/} (Bi et al., 2021, Canon et al., 2020, Chen et al., 2020, Couturier et al., 2020, Darmanis et al., 2017, Hovestadt et

al., 2019, Hwang et al., 2022, Ji et al., 2020, Kürten et al., 2021, Ma et al., 2019, Neftel et al., 2019, Pelka et al., 2021, Qian et al., 2020, Rendeiro et al., 2020, Riether et al., 2020, Wang et al., 2019, Wu et al., 2020, Zhou et al., 2021), The UCSC Cell Browser \url{https://cells-test.gi.ucsc.edu/?ds=early-brain} (Eze et al., 2021), GEO under accession number GSE173278 [\url{https://www.ncbi.nlm.nih.gov/geo/query/acc.cgi?acc=GSE173278}](Leblanc et al., 2022), GSE193884[\url{https://www.ncbi.nlm.nih.gov/geo/query/acc.cgi?acc=GSE193884}](Leblanc et al. 2022), GSE129730[\url{https://www.ncbi.nlm.nih.gov/geo/query/acc.cgi?acc=GSE129730}](Ocasio et al., 2019), GSE122871[\url{https://www.ncbi.nlm.nih.gov/geo/query/acc.cgi?acc=GSE122871}](Weng et al., 2019), GSE156633[\url{https://www.ncbi.nlm.nih.gov/geo/query/acc.cgi?acc=GSE156633}](Luo et al., 2021), The Neuroblastoma Cell Atlas \url{https://www.neuroblastomacellatlas.org/} (Kildisiute et al., 2021, ArrayExpress under accession number E-MTAB-9296[\url{https://www.ebi.ac.uk/biostudies/arrayexpress/studies/E-MTAB-9296}](Larsson et al., 2021), The Sequence Read Archive under accession number PRJNA637987[\url{https://www.ncbi.nlm.nih.gov/bioproject/PRJNA637987/}](Manno et al., 2021). Additional details about the publicly available datasets can be found in Table 1. Figure data can be found at \url{https://doi.org/10.6084/m9.figshare.25909156}. Source data are provided with this paper.

Research involving human participants, their data, or biological material

Policy information about studies with human participants or human data. See also policy information about sex, gender (identity/presentation), and sexual orientation and race, ethnicity and racism.

Reporting on sex and gender	The study involves an experiment on 6 primary human cell lines. The cell lines were chosen based on their MGMT methylation status, sex was not the primary consideration in the study design, but the six cell lines are derived from both male (n=4) and female (n=2) patients.
Reporting on race, ethnicity, or other socially relevant groupings	N/A
Population characteristics	N/A
Recruitment	N/A
Ethics oversight	Primary glioblastoma cell lines were used in this study, as detailed in section "Knockdown experiments" and "Drug combination treatments". These cell lines were obtained from the human glioblastoma cell culture (HGCC) resource. For the establishment of the HGCC resource (previous work, not done during this study), tumor sample collection was approved by the Uppsala Regional Ethical Review Board number 2007/353. Further details on patient informed consent and compensation can be found at https://www.hgcc.se/#.

Note that full information on the approval of the study protocol must also be provided in the manuscript.

Field-specific reporting

Please select the one below that is the best fit for your research. If you are not sure, read the appropriate sections before making your selection.

☒ Life sciences ☐ Behavioural & social sciences ☐ Ecological, evolutionary & environmental sciences

For a reference copy of the document with all sections, see nature.com/documents/nr-reporting-summary-flat.pdf

Life sciences study design

All studies must disclose on these points even when the disclosure is negative.

Sample size	All data analysis was performed on single-cell RNA sequencing data, where the sample number is inherently high. Model robustness and clustering stability was evaluated through an extensive simulation study, as outlined in Appendix.<br><br>The drug combination treatments and CRISPR-KD experiments contained replicates, as described in Methods.
Data exclusions	In the processing of the downloaded single-cell RNA sequencing data, a QC step filtered out cells containing less than 200 genes and genes present in less than 3 cells. In addition, all non-malignant cells were filtered out according to the metadata annotations.
Replication	The drug combination treatments and CRISPR-KD experiments contained replicates, as described in Methods. All replicate measurements are reported in the manuscript and replication of biological findings were successful.
Randomization	In the drug treatment experiment a plate layout randomization protocol was employed to avoid any spatial bias to impact experimental results. For the comparison between MGMT methylated and unmethylated cell lines, samples were allocated to groups based on their MGMT methylation status. When choosing cell lines, covariates such as sex and subtype were taken into consideration for an even representation when possible.
Blinding	Investigators were not blinded to group allocation. Blinding could not be done as it was the same researcher who chose the cell lines for experiments, conducted the experiments and analyzed the experiment.

Reporting for specific materials, systems and methods

We require information from authors about some types of materials, experimental systems and methods used in many studies. Here, indicate whether each material, system or method listed is relevant to your study. If you are not sure if a list item applies to your research, read the appropriate section before selecting a response.

Materials & experimental systems

n/a	Involved in the study
☒	☐ Antibodies
☐	☒ Eukaryotic cell lines
☒	☐ Palaeontology and archaeology
☒	☐ Animals and other organisms
☒	☐ Clinical data
☒	☐ Dual use research of concern
☒	☐ Plants

Methods

n/a	Involved in the study
☒	☐ ChIP-seq
☒	☐ Flow cytometry
☒	☐ MRI-based neuroimaging

Eukaryotic cell lines

Policy information about cell lines and Sex and Gender in Research

Cell line source(s)	Primary cell lines U3013MG (F, age 78), U3017MG (M, age 68), U3028MG (F, age 72), U3065MG (M, age 77), U3071MG (M, age 65) and U3180MG (M, age 77) were provided by the original source of cell line development (hgcc.org).
Authentication	Primary cells were provided from the original source. No further authentication of the cell lines was performed.
Mycoplasma contamination	All cell lines tested negative for mycoplasma contamination.
Commonly misidentified lines (See ICLAC register)	No commonly misidentified cell lines were used in the study.

Plants

Seed stocks	N/A
Novel plant genotypes	N/A
Authentication	N/A
